# Anti-Allergic Effect of Dietary Polyphenols Curcumin and Epigallocatechin Gallate via Anti-Degranulation in IgE/Antigen-Stimulated Mast Cell Model: A Lipidomics Perspective

**DOI:** 10.3390/metabo13050628

**Published:** 2023-05-05

**Authors:** Jun Zeng, Jingwen Hao, Zhiqiang Yang, Chunyu Ma, Longhua Gao, Yue Chen, Guiling Li, Jia Li

**Affiliations:** 1 College of Ocean Food and Biological Engineering, Jimei University, Xiamen 361021, China; 2 Xiamen Key Laboratory of Marine Functional Food, Xiamen 361021, China; 3 The Affiliated Stomatology Hospital, School of Medicine, Zhejiang University, Hangzhou 310000, China; 4 Key Laboratory of Tea Biology and Resources Utilization, Ministry of Agriculture, Tea Research Institute, Chinese Academy of Agricultural Sciences, Hangzhou 310008, China

**Keywords:** allergy, dietary polyphenols, curcumin, EGCG, lipidomics

## Abstract

Polyphenol-rich foods exhibit anti-allergic/-inflammatory properties. As major effector cells of allergies, mast cells undergo degranulation after activation and then initiate inflammatory responses. Key immune phenomena could be regulated by the production and metabolism of lipid mediators by mast cells. Here, we analyzed the antiallergic activities of two representative dietary polyphenols, curcumin and epigallocatechin gallate (EGCG), and traced their effects on cellular lipidome rewiring in the progression of degranulation. Both curcumin and EGCG significantly inhibited degranulation as they suppressed the release of β-hexosaminidase, interleukin-4, and tumor necrosis factor-α from the IgE/antigen-stimulated mast cell model. A comprehensive lipidomics study involving 957 identified lipid species revealed that although the lipidome remodeling patterns (lipid response and composition) of curcumin intervention were considerably similar to those of EGCG, lipid metabolism was more potently disturbed by curcumin. Seventy-eight percent of significant differential lipids upon IgE/antigen stimulation could be regulated by curcumin/EGCG. LPC-O 22:0 was defined as a potential biomarker for its sensitivity to IgE/antigen stimulation and curcumin/EGCG intervention. The key changes in diacylglycerols, fatty acids, and bismonoacylglycerophosphates provided clues that cell signaling disturbances could be associated with curcumin/EGCG intervention. Our work supplies a novel perspective for understanding curcumin/EGCG involvement in antianaphylaxis and helps guide future attempts to use dietary polyphenols.

## 1. Introduction

Allergic diseases, including food allergies, allergic asthma, rhinitis, and dermatitis, cause significant morbidity worldwide [1,2]. Food allergies, for instance, occur in up to 10% of the worldwide population and are associated with an increasing prevalence every year [2]. Allergy is a serious public health and food safety concern. 

Dietary components have gained increasing attention in recent years for their ability to prevent and alleviate allergic responses [2,3]. As the main component in fruits, vegetables, and other edible plant parts, polyphenols and polyphenol-rich foods have been reported to exhibit anti-allergic/-inflammatory properties [2,3]. Dietary polyphenols, such as curcumin (turmeric) and epigallocatechin gallate (EGCG, green tea), are emerging topics of interest and research, as these compounds exhibit potential benefits for human health [4]. Polyphenols may interfere with allergic reactions by inhibiting the release of chemical mediators (histamine, hexosaminidase, or leukotrienes), cytokine production, signal transduction, and gene expression in mast cells, basophils, or T cells [2]. Furthermore, the formation of complexes between polyphenols and proteins, such as peanut and cashew proteins, has been shown to prevent antibody recognition of allergens via allergen precipitation and reduce IgE binding to allergens [5,6,7]. Plant-based proteins (PP)–phenolic compounds (PC) conjugates and complexes have been reported to exhibit potential allergy-reducing activities [8]. The regulation of gut microbiota by polyphenols might also contribute to their anti-allergic/-inflammatory properties [2]. An increasing number of clinical and epidemiological studies have provided evidence that dietary polyphenol consumption and a reduction in risk factors for chronic allergic diseases are correlated [3]. Although in vivo and in vitro studies have indicated polyphenols’ anti-allergic/-inflammatory effects, knowledge of their mechanism of action remains incomplete. A deeper understanding of pathogenic mechanisms is needed to explore promising biomarkers of allergic diseases as well as identify the involvement of polyphenols in cellular and molecular events central to antianaphylaxis. 

The immune mechanism underlying allergic disorders encompasses an adaptive Th2-type response [2]. Antigen-specific immunoglobulin E (IgE) antibodies, together with major effector cells of allergies (i.e., the mast cell), are crucial for the development of allergic disorders [9]. Mast cells express the high-affinity IgE receptor FcεRI on their surface [10]. Antigen stimulation activates mast cells sensitized with IgE [1]. Mast cells undergo degranulation after activation, initiate an acute inflammatory response, and contribute to the progression of chronic diseases [1]. The degranulation of mast cells results in the release of β-hexosaminidase, a common degranulation marker, histamine, inflammatory cytokines, and lipid-derived mediators [10]. Mast cells are well-known producers of different lipid mediators [9]. Currently, the production and metabolism of these lipid mediators have in turn been shown to regulate mast cell functions in an increasing number of studies [9,11]. Bioactive lipids of leukotrienes, prostanoids, platelet-activating factor (PAF), and sphingolipids have been reported to influence cell signaling via multiple mechanisms [12], including by formatting structural support platforms (lipid rafts) for receptor signaling complexes, by transducing signals as primary/secondary messengers, and by serving as kinase/phosphatase cofactors [12]. Then, bioactive lipids could remodel key immune phenomena (degranulation, chemotaxis, and sensitization). In rapidly emerging research, the modulation of mast cell reactivity by lipid metabolism, in addition to proteins, is revealing novel and unprecedented targets [9]. These targets may serve to preclude mast cell effects in allergic reactions. Thus, it is important to gain more comprehensive insights into the effects of lipidome remodeling on mast cell degranulation, changes in cellular lipid composition induced by allergens, and changes in lipid transport and metabolism in mast cells. High-throughput lipidomics is an emerging analytical strategy that enables a wide range of lipids to be explored on the scale of individual lipid molecular species, supplying a global and detailed map of the lipidome response to external stimulation [13]. To the best of our knowledge, on the scale of the lipidome, the characterization of mast cell lipid composition and stimulus-specific changes upon polyphenol interventions remains limited.

Turmeric, the major source of curcumin, is a spice that has been traditionally used in Asian countries for culinary purposes. As a natural polyphenol derivative, curcumin got approved to be “generally recognized as safe” (GRAS) by the US Food and Drug Administration (FDA) [14]. The tea plant (*Camellia sinensis*) is native to East Asia and has traditionally been consumed worldwide as “tea”. Green tea is also a rich source of the natural polyphenol EGCG, which is present most abundantly [15]. Curcumin and EGCG were suggested to exhibit anti-allergic/-inflammatory potential in previous reports [3,14] and our preliminary studies. The compounds were defined as two typical dietary polyphenols in this study to analyze and compare their mechanisms of action underlying the progression of mast cell degranulation. As an in vitro mast cell model, the basophilic leukemia (RBL-2H3) cell line has been successfully applied in previous studies to investigate IgE-FcεRI interactions and degranulation, as well as screen antiallergy drug candidates [16,17]. According to previous studies that traced the release of mediators from mast cells, the allergic reaction progresses along the following time course: the immediate phase (within 1 h of allergen challenge) and the later phase (after 3–48 h) [18]. Hence, in this study, curcumin/EGCG intervention in the progression of degranulation was explored at the interfacial stage of the allergic reaction (i.e., 1 h and 3 h). Specially, we investigated comprehensive lipidome rewiring in an IgE/antigen (i.e., dinitrophenyl-bovine serum albumin, DNP-BSA)-stimulated RBL-2H3 degranulation model using a nontargeted lipidomics approach based on ultra-high-performance liquid chromatography coupled to mass spectrometry (UPLC—MS). A sample set of cells from the control groups and curcumin/EGCG intervention groups was traced at both 1 h and 3 h for lipidomics investigation. Our work provides a novel perspective on understanding the action of antigen stimulation and curcumin/EGCG involvement in antianaphylaxis.

## 2. Materials and Methods

### 2.1. Cell Culture and Cell Viability

RBL-2H3 cells were purchased from Procell Life Science & Technology Co., Ltd. (Wuhan, China) and cultured in minimal essential medium (MEM) with 15% heat-inactivated fetal bovine serum (FBS), 100 units/mL penicillin, and 100 μg/mL streptomycin at 37 °C in a humidified incubator (5% CO_2_). The cytotoxic effects of curcumin and EGCG were evaluated by MTT assay (CellTiter 96 Aqueous One Solution Cell Proliferation Assay; Promega, Solarbio, Beijing, China) (n = 6).

### 2.2. Sample Collection

Chemicals and reagents are provided in the Supporting Information. First, RBL-2H3 cells were incubated with 200 ng/mL anti-DNP-IgE for 18 h. After washing with phosphate-buffered saline (PBS) three times, the IgE-sensitized cells were exposed to 10 μM curcumin (i.e., Cur group) or 200 μM EGCG (i.e., EGCG group) and then stimulated with 500 ng/mL DNP-BSA simultaneously. The coincubation of IgE-sensitized cells with curcumin/EGCG and DNP-BSA was performed for 1 h or 3 h. A vehicle control group without both IgE and DNP-BSA was set up in parallel with the experiment (i.e., Veh group), as well as a positive control group prepared by incubation of IgE-sensitized cells with the DNP-BSA antigen (i.e., AG group). 

**Figure 1:**
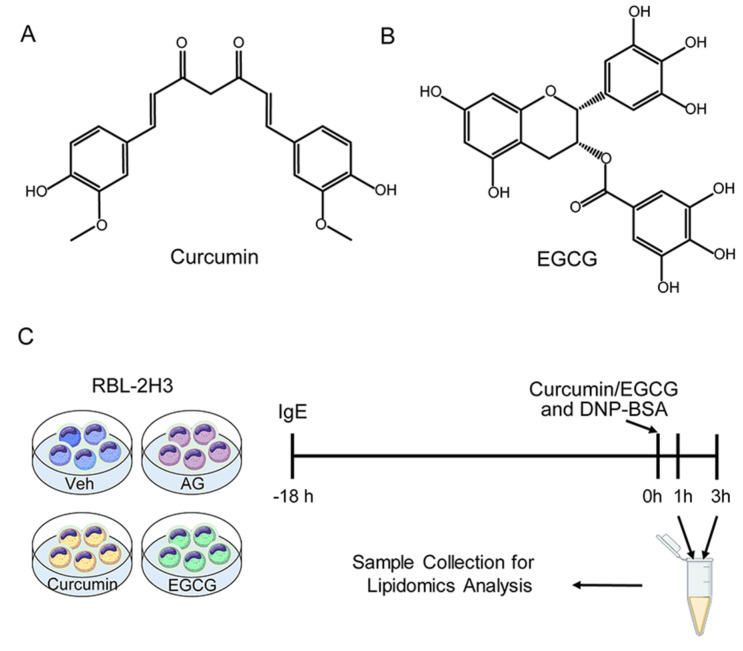
Characteristics of the experimental model. (**A**) Structure of curcumin. (**B**) Structure of EGCG. (**C**) Scheme of the experimental design. Both curcumin and EGCG inhibit IgE-mediated degranulation in the RBL-2H3 cell model. Veh: vehicle control group; AG: IgE/antigen stimulation group; curcumin: curcumin intervention group; EGCG: EGCG intervention group.

The structures of curcumin and EGCG and the scheme of this experimental design are shown in Figure 1A–C. To monitor the curcumin/EGCG intervention on IgE-mediated degranulation, cell specimens from control groups (incl. Veh and AG) and intervention groups (incl. Cur and EGCG) were collected at both 1 h and 3 h. Four independent biological replicates of RBL-2H3 cells were prepared for each group at each time point for the lipidomics study.

### 2.3. Nontargeted Lipidomics Study

Each sample was placed in a 10 cm Petri dish with 8 mL of cell media (about 10^7^ cells). The cell medium was completely aspirated after sample collection, followed by washing with Dulbecco’s phosphate-buffered saline (DPBS) solution and inactivation with liquid nitrogen immediately. 

After lipidome extraction, freeze-dried lipid extracts were reconstituted in 10 μL of dichlormethane:methanol (2:1) and then diluted five times in ACN:isopropanol:water (65:30:5). Finally, 50 μL of cell lipid extracts was analyzed using an UltiMate 3000 UPLC system (Thermo, Waltham, MA, USA) coupled with a quadrupole Orbitrap mass spectrometer (Q-Exactive, Thermo, USA). LC separation was performed using a BEH C8 column (2.1 × 100 mm, 1.7 μm) (Waters, Milford, MA, USA). Full-scan MS for lipid profiling and data-dependent MS/MS (ddMS2) for lipid identification were performed in both positive and negative electrospray (ESI) ion modes. 

Details of the lipidomics study, including lipidome extraction and lipidomics analysis by UPLC-Q-Exactive MS, are provided in the Supporting Information and were adopted from our previously published method [19,20,21]. Quality control (QC) samples, which were generated by pooling equal aliquots of lipid extracts from each sample, were prepared as real samples and regularly inserted into the analysis sequence to monitor the robustness of lipidomic analysis.

### 2.4. Data Processing and Statistics

Lipid species were identified according to accurate *m/z*, tandem mass spectrometry (MS/MS) fragmentation patterns, and retention behavior. The LIPID MAPS database (http://www.lipidmaps.org/, accessed on 1 January 2023) and MS-DIAL software (http://prime.psc.riken.jp/compms/msdial/main.html, accessed on 1 January 2023) were used for lipid queries. For the quantification of these identified lipids, peak areas were obtained by high-resolution extracted ion chromatogram using Trace Finder software (Thermo, USA). Two thresholds, both *m/z* and retention time, were applied to the extraction process of peak area (an *m/z* tolerance of ±5 ppm and a retention time extraction window of ± 15 s). Peak checking and noise removal were carried out to reduce errors. 

To eliminate systematic bias, the peak area of each lipid species was normalized to the total intensity of all lipid species in a given sample. Prior to statistical analysis, lipids with a percentage relative standard deviation (%RSD) higher than 30% in all QCs were removed from the dataset. Then, the dataset was subjected to SIMCA-P 11.0.0.0 software (Umetrics, Malmö, Sweden) for principal component analysis (PCA) and partial least squares discriminant analysis (PLS-DA) with unit variance (UV) scaling. To assess the univariate statistical significance, two-way analysis of variance (ANOVA), Wilcoxon Mann—Whitney test, and false discovery rate (FDR) correction (Benjamini—Hochberg method) were employed using Multi Experiment Viewer (MeV) software (open-source genomic analysis software, version 4.9.0) and an in-house-developed MATLAB program (The MathWorks, Natick, MA, USA). On the basis of hierarchical cluster analysis (HCA), a heatmap was also generated with MeV to visualize the relative levels of lipids. Receiver operating characteristic curve (ROC) and binary logistic regression were performed using SPSS Statistics software (SPSS Inc., Chicago, DE, USA). 

The abbreviations used in this study for lipid classes are as follows: (Hex)Cer, (hexosyl)ceramide; SM, sphingomyelin; CE, cholesterol esters; (L)PC, (lyso)phosphatidylcholine; (L)PE, (lyso)phosphatidylethanolamine; OxPE, oxidized phosphatidylethanolamine; (L)PG, (lyso)phosphatidylglycerol; (L)PI, (lyso)phosphatidylinositol; (L)PS, (lyso)phosphatidylserine; (L)PA, (lyso)phosphatidic acid; PEtOH, phosphatidylethanol; DG, diacylglycerol; TG, triacylglycerol; CL, cardiolipin; CoQ, coenzyme Q; ASM, acylsphingomyelin; NAE, N-acyl ethanolamines; GM3, ganglioside GM3; FA, fatty acid; OxFA, oxidized fatty acid; CAR, acylcarnitine; (H)BMP, (hemi)bismonoacylglycerophosphate; ST, sterol; o/p-, ether and plasmalogen.

### 2.5. β-Hexosaminidase Release Assay

β-Hexosaminidase activity in culture supernatants was measured as an indicator of degranulation [10]. The amount of β-hexosaminidase released from RBL-2H3 cells was quantified according to previous reports with slight modifications (n = 3) [22]. 

Briefly, after the coincubation of IgE-sensitized cells with curcumin/EGCG and DNP-BSA, the supernatant was collected and centrifuged at 1500 rpm for 5 min, while the cells were incubated in Tyrode’s buffer containing 1% Triton X-100 for 5 min. The supernatant and cell lysate were transferred to 96-well black microplates (25 μL/well) and then incubated with 1.2 mM 4-methylumbelliferyl-N-acetyl-β-D-glucosamincide dissolved in 0.1 M citrate buffer (pH 4.5) at 37 °C for 30 min (100 μL/well). The fluorescence intensity was measured at 450 nm with a microplate reader. The β-hexosaminidase release (%) and inhibition of release (%) were calculated as follows:

(1) $$\beta \mathrm{hexosaminidase}\;\mathrm{release}\left(\%\right)=\frac{F_{\mathrm{supernatant}}}{F_{\mathrm{supernatant}}+F_{\mathrm{cell}\;\mathrm{lysate}\;}}\times 100$$

(2) $$\mathrm{Inhibition}\;\mathrm{of}\;\mathrm{release}\;(\%)=\frac{\mathrm{AG}-\mathrm{Intervention}}{\mathrm{AG}-\mathrm{Veh}}\times 100$$

where F in Equation (1) is the fluorescence intensity. AG, Veh, and intervention in Equation (2) refer to the β-hexosaminidase release (%) of the AG, Veh, and intervention groups. 

### 2.6. TNF-α and IL-4 Release Assay

To determine the tumor necrosis factor-α (TNF-α) and interleukin-4 (IL-4) concentrations in the culture media, all samples were centrifuged (17,000× *g*, 10 min) at 4 °C and stored at −80 °C until analysis. Then, the levels of TNF-α and IL-4 were measured using ELISA kits (Elabscience, Wuhan, China), in accordance with the manufacturers’ instructions (n = 3).

## 3. Results

### 3.1. Inhibitory Effect of Curcumin/EGCG on IgE-Mediated Degranulation

**Figure 2:**
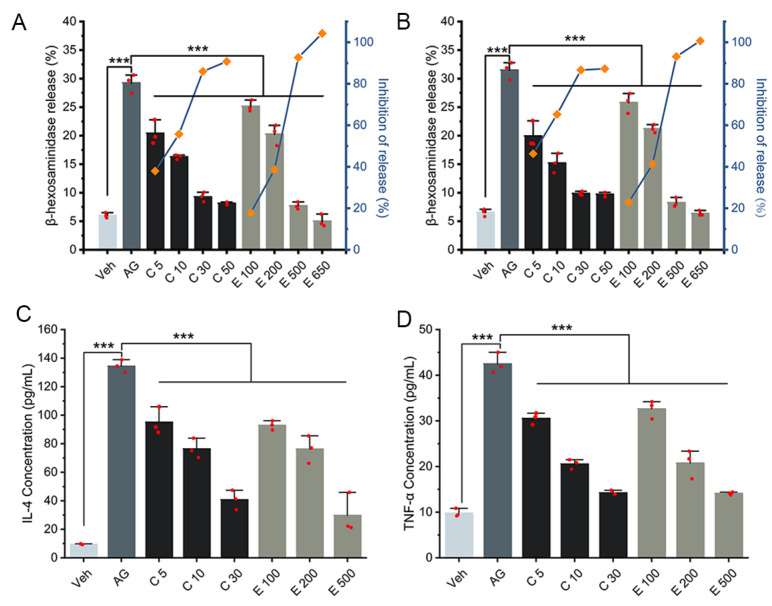
Both curcumin and EGCG inhibit IgE-mediated degranulation in the RBL-2H3 cell model. (**A**,**B**) exhibit β-hexosaminidase release at 1 h and 3 h, respectively. The β-hexosaminidase release (%) is represented by each column, and the line reveals the inhibition of release (%). (**C**) IL-4 release at 3 h. (**D**) TNF-α release at 3 h. All data are presented as the mean ± SD. Individuals in each group are represented by the red dot in each column. C5, C10, C30, and C50 denote intervention groups in which IgE/antigen-stimulated cells were treated with 5, 10, 30 and 50 μM curcumin; E100, E200, E500, and E650 denote intervention groups in which IgE/antigen-stimulated cells were treated with 100, 200, 500, and 650 μM EGCG. ***: *p* < 0.001.

The release of β-hexosaminidase was first measured as a general indicator of degranulation and a hallmark characteristic of allergic reactions upon allergen stimulation [10]. The modeling with 200 ng/mL anti-DNP-IgE for sensitization and the coincubation of DNP-BSA and curcumin/EGCG for stimulation was defined in our preliminary studies for better inhibition of β-hexosaminidase release (Figure 2A,B and Figure S1A–C). 

To assess the effect of curcumin/EGCG on the IgE-mediated allergic response, cell morphology and the release of two representative proinflammatory cytokines (TNF-α and IL-4) were also analyzed, in accordance with the evaluation of β-hexosaminidase release. RBL-2H3 cells from the Veh group displayed fibroblastic morphology (Figure S2). Activating RBL-2H3 cells by an IgE–antigen complex induced cell swelling, and significantly improved the levels of β-hexosaminidase, IL-4, and TNF-α (Figure S2 and Figure 2). After curcumin/EGCG treatment, the activated cells exhibited improved morphology, and the release of β-hexosaminidase, IL-4, and TNF-α was significantly suppressed in a dose-dependent manner (Figure S2 and Figure 2).

Cell viability was not obviously affected at concentrations less than 10 μM curcumin and 200 μM EGCG (Figure S1D,E). Then, 10 μM curcumin and 200 μM EGCG were used in subsequent lipidomics studies. We observed that the suppression of β-hexosaminidase release by 10 μM curcumin (i.e., 55.72% and 65.27% inhibition of release for 1 h and 3 h, respectively) was more potent than that by 200 μM EGCG (i.e., 38.61% and 41.24% inhibition of release for 1 h and 3 h, respectively; Figure 2A,B). Compared with that at 1 h, the percent inhibition of β-hexosaminidase release at 3 h increased, indicating the progression of curcumin/EGCG intervention. Interestingly, the decrease in proinflammatory cytokine production induced by 10 μM curcumin and 200 μM EGCG was similar. These results confirmed that the inhibition of IgE-mediated RBL-2H3 degranulation by curcumin/EGCG was successfully implemented in this study.

### 3.2. Lipidome of RBL-2H3 Cells

To trace curcumin/EGCG action in the progression of degranulation, RBL-2H3 cells from the control groups (incl. Veh and AG) and intervention groups (incl. Cur and EGCG) were analyzed at both 1 h and 3 h for lipidomics investigation. The large-scale lipidomics profiling of RBL-2H3 cells revealed approximately 1800 lipid features in a nontargeted pattern (Figure S3). A total of 957 lipid species were finally identified, including 75 fatty acyls, 161 glycerolipids, 582 glycerophospholipids, 123 sphingolipids, 14 sterols, and 2 prenols (Tables S1, S2 and Figure S4). This profiling revealed that the chemical structures, compositions, and polarities of the cellular lipidome were largely diverse and complex.

The reliability and robustness of the acquired lipidomics data were investigated by evaluating QC samples and confirmed to be satisfactory for complex biological samples (Figure S5). Detailed information on the identified lipids and QC evaluation is described in the Supporting Information.

### 3.3. Global Profiling of Lipidome Disturbance

**Figure 3:**
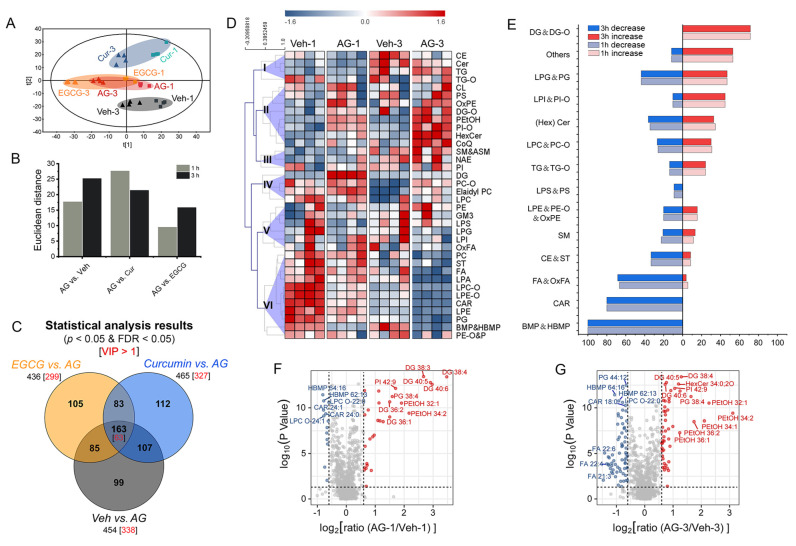
Global profiling of lipidome disturbance and lipidome changes associated with IgE-mediated degranulation. In lipidomics sampling, 10 μM curcumin and 200 μM EGCG were defined based on cell viability results. (**A**) PCA score plot for the classification of vehicle (Veh-1 and Veh-3), IgE/antigen stimulation (AG-1 and AG-3), curcumin (Cur-1 and Cur-3), and EGCG (EGCG-1 and EGCG-3) groups at both 1 h and 3 h. (**B**) Euclidean distance. (**C**) Venn diagram for an overview of the statistical results. The black numbers mean lipids with *p* < 0.05 and FDR < 0.05. The red numbers mean lipids with *p* < 0.05, FDR < 0.05, and VIP > 1. (**D**) Heatmap of each lipid class. The sum of the relative responses from each lipid class was UV scaled and subjected to hierarchical clustering. (**E**) Percentage of significantly differential lipids in response to IgE-mediated degranulation (Veh vs. AG, *p* < 0.05 and FDR < 0.05). The number of lipid species that were either significantly up- or downregulated was normalized to the total number of lipids detected in each family. (**F**,**G**) volcano plots for the comparisons at 1 h and 3 h, respectively. The red (or blue) dot denotes the lipid with *p* < 0.05 and a ratio more than 3/2 (or less than 2/3).

Global profiling of the cellular lipidome was visualized by unsupervised PCA. Two types of metabolic disturbance, time-related and treatment-induced changes, were clearly visible on the score plot (Figure 3A). Cell specimens collected at 1 h and 3 h were presented on the two sides of the PCA score plot. The control (i.e., Veh and AG) and different intervention groups (i.e., Cur and EGCG) showed a clear trend of discrimination along the second principal component. An overview of these lipidome differences was further quantified by analyzing the Euclidean distance between the AG group and other groups at each time point (Figure 3B). 

To specify significant differential lipids in response to allergen stimulation and curcumin/EGCG intervention, the univariate statistical significance of lipids was evaluated (*p* < 0.05 and FDR < 0.05). Three PLS-DA models (i.e., Veh vs. AG, curcumin vs. AG, and EGCG vs. AG, Figures S6–S8) were developed to screen significant differential lipids based on variable importance in the projection (VIP) values (VIP > 1). Lipids with multivariate and univariate statistical importance in the classification were cross-refined (i.e., the intersection, Figure 3C) and then assumed to be representative differential characteristics.

### 3.4. Lipidome Changes Associated with IgE-Mediated Degranulation

The comparison between the AG and Veh groups indicated that the cellular lipid metabolism was rewired during the progression of IgE-mediated degranulation (Figure 3A and Figure S6). The classification of PCA showed robust lipidome disturbances in response to allergen stimulation at both 1 h and 3 h (Figure 3A), and the progression of degranulation was supported by the more evident metabolic changes at 3 h when compared with 1 h (Figure 3B). A total of 454 significant differential lipids changed by degranulation were discovered (Veh vs. AG, *p* < 0.05 and FDR < 0.05, Figure 3C, Table S3).

The sum of responses in each lipid class was analyzed to cluster into six major groups in a heatmap according to the similarity of variation tendencies (Figure 3D). Enrichment of changes in lipid classes was pinpointed by normalizing the number of significant differential lipids changed by degranulation to the total number of lipids detected in each family (Figure 3E). DG(-O) (incl. DG and DG-O), manifesting a greater response in AG at both 1 h and 3 h, were found to be the top lipid category associated with significant upregulation upon allergen stimulation (Figure 3E). In contrast, (H)BMP (incl. BMP and HBMP), CAR, and (Ox)FA (incl. FA and OxFA) were discovered to be the top lipid classes associated with significant downregulation in AG (Figure 3D,E). Notably, the percentage of either increased or decreased lipids with significant differences was similar between 1 h and 3 h (Figure 3E), indicating that common lipidome change patterns are shared during the progression of degranulation.

Unique metabolic disturbances were also observed at different stages of degranulation. Volcano plots indicated that at the later stage of 3 h, the fold change of significant differential lipids increased and the range of fold changes broadened, suggesting that the metabolic disturbances progressed (Figure 3F,G). Specifically, DGs were observed with an obvious increase at 1 h, followed by a callback at 3 h (Figure 3F). With the progression of degranulation, FAs underwent downregulation from the immediate phase to the later phase and exhibited a prominent decrease upon 3 h of antigen stimulation (Figure 3G). The emergence of these response patterns may involve degranulation dynamics.

### 3.5. Comparison between Curcumin and EGCG Intervention

Global profiling of the cellular lipidome was analyzed to depict the intervention effects of curcumin/EGCG from the perspective of lipidome remodeling (Cur vs. AG and EGCG vs. AG, Figures S7 and S8). 

Multivariable differences were pinpointed by Euclidean distances in PCA (Figure 3B). Unlike the EGCG group, curcumin intervention was evidenced by the more evident metabolic changes at 1 h compared with 3 h, highlighting the active intervention by curcumin at the early stage of allergen stimulation. Univariate statistical tests identified 454 significant differential lipids in response to allergen actions (Table S3). According to the Venn diagram (Figure 3C), a total of 270 (59%) and 248 (55%) of these differential lipids exhibited significant quantitative alterations when the curcumin and EGCG groups were compared with the AG group (*p* < 0.05 and FDR < 0.05). Together, 355 (78%) of the significant differential lipids could be changed upon either curcumin or EGCG intervention. Both multivariable and univariate statistical results implied that the lipidome intervention in Cur was more prominent than that in EGCG, which is consistent with their differences in the suppression of IgE-mediated RBL-2H3 degranulation (Figure 2). 

**Figure 4:**
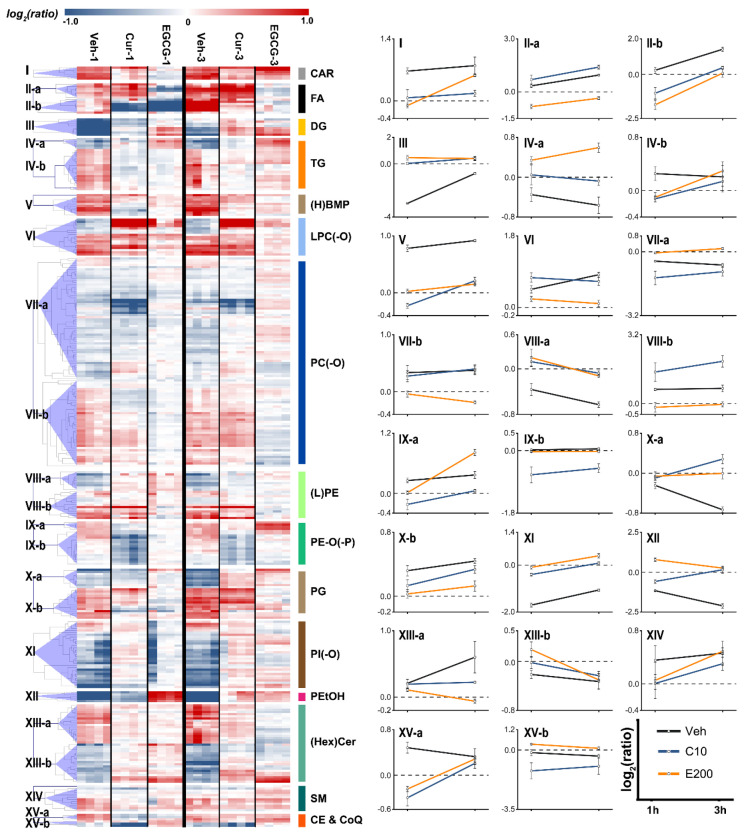
Comparison between curcumin and EGCG intervention. Representative differential lipids associated with allergen stimulation were subjected to heatmap to specify the regulation by curcumin/EGCG. The conversion dataset with relative contents of lipids (i.e., the contents of lipids for each sample divided by the average values from time-matched AG individuals) was logarithmically scaled, and then categorized in the tree of hierarchical clustering analysis based on the similarity of the regulation-response pattern (**left** panel). Representative metabolites were selected from each cluster to present the response trajectory (**right** panel). Each point in the trajectory was presented as the average relative content ± SD.

To specify the lipidome regulation by curcumin/EGCG, the representative differential lipids associated with allergen stimulation were further subjected to a heatmap (Figure 4). The baseline level was defined as the average readings from time-matched AG groups. The lipid contents of each sample from the Veh, Curcumin, and EGCG groups were then divided by the average of time-matched AG groups to produce the ratio. As compared to the alteration pattern associated with allergen stimulation (Veh vs. AG), the lipidome modulation by curcumin/EGCG could be identified (Cur vs. AG and EGCG vs. AG). Both curcumin and EGCG regulate the response pattern of CAR, CE, CoQ, glycerophospholipids [(H)BMP, LPC(-O) (incl. LPC and LPC-O), PC(-O) (incl. PC and PC-O), PG, (L)PE (incl. PE and LPE) and PE(-O/-p)], and sphingolipids [(Hex)Cer (incl. Cer and HexCer) and SM] at either 1 h or 3 h. These lipid species exhibited similar response patterns between the intervention and Veh groups, implying their sensitivity to both curcumin and EGCG interventions. Aside from those common features, curcumin exhibits a greater potency than that of EGCG in the upregulation of FA metabolism. Curcumin recovered the abundance of DG and PEtOH with an immediate decrease at 1 h. In contrast, the abundance of CAR and TG was improved by EGCG at 3 h. Special nonrecovered response patterns were also discovered for the curcumin/EGCG intervention groups, in which the changes in lipids were different from those of the control group. The inherent biological variation could at least partially contribute to the emergence of these response patterns. Another explanation might involve the functional diversity of polyphenols. 

**Figure 5:**
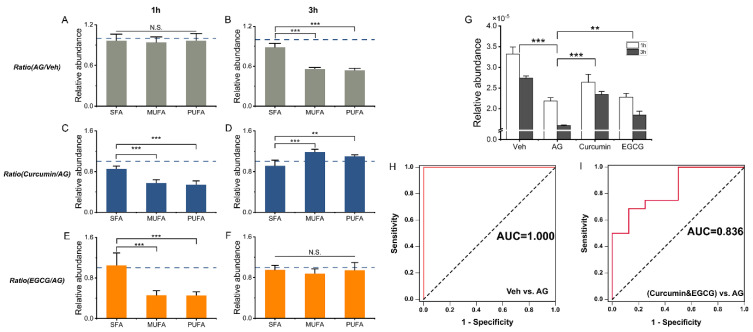
Changes in the content and composition of important lipids. (**A**–**F**) present FA changes associated with acyl chain composition. (**G**) Relative content of LPC-O 22:0. (**H**,**I**) ROC curves of LPC-O 22:0. Diagnostic potential was evaluated based on binary logistic regression. Each column is presented as the mean ± SD. **: 0.001 < *p* < 0.01, ***: *p* < 0.001, N.S.: no significance.

We further determined whether there were any changes associated with the lipid composition upon curcumin/EGCG intervention (Figure 5A and Figure S9). At the immediate phase of allergen stimulation, no significant alterations in FA acyl chain composition were observed (Figure 5A). After 3 h of allergen stimulation (i.e., the later phase), there was a significantly lower abundance of unsaturated fatty acids [incl. monounsaturated fatty acids (MUFAs) and polyunsaturated fatty acids (PUFAs)] than saturated fatty acids (SFAs) (Figure 5B). Three hours of curcumin intervention significantly improved the depletion of MUFA/PUFA (*p* < 0.05, Figure 5C,D). Likewise, the MUFA/PUFA content was greatly replenished by 3 h of EGCG treatment (Figure 5E,F). 

Then, from the perspective of lipidome remodeling, the activity of curcumin/EGCG in the suppression of IgE-mediated degranulation is involved in the comprehensive regulation of both lipid response and composition. 

### 3.6. Defining Potential Biomarkers

Considering the association between phenotype and metabolic changes, we further examined potential biomarkers to discriminate degranulation and validate curcumin/EGCG inhibition.

Compared with the control group, allergen stimulation induced a total of 338 representative significant differential lipids that were cross-refined by screening for univariate statistical significance (*p* < 0.05, FDR < 0.05), multivariate VIP values (VIP > 1), and covariance *p(corr)* values (|*p(corr)*| > 0.3). These significant lipids were further picked via the criteria of change magnitude (fold change >3/2 or <2/3) and stricter analysis quality (within-group variation < 15%). Then, these candidates were subjected to ROC analysis. A total of 19 lipids were finally designated as biomarker candidates with the best discrimination ability (AUC = 1), spanning the lipid categories of CAR, DG, HBMP, PEtOH, PG, PI(-O), and LPC-O (Table 1).

The biomarker candidates that were sensitive to allergen stimulation were subsequently validated in the evaluation of the curcumin/EGCG intervention. Curcumin significantly recovered the abundance of LPC-O 22:0, LPC-O 24:1, HBMP 58:8, CAR 24:0, CAR 24:1, and PG 38:4, while LPC-O 22:0 could be significantly improved by EGCG. LPC-O 22:0 was discovered in the overlap.

LPC-O 22:0 was defined as a potential biomarker (Figure 5G and Figure S10). The exploitation of LPC-O 22:0 achieved an AUC value of 1 in the discrimination of degranulation (Veh vs. AG), and both the sensitivity and specificity were 100% (Figure 5H). Furthermore, for the comparison between all intervention individuals (incl. curcumin and EGCG intervention groups) and AG groups, satisfactory discrimination results were also acquired in the evaluation of inhibition manner, resulting in an AUC value of 0.836 and sensitivity and specificity of 68.8% and 87.5%, respectively (Figure 5I). These evaluations confirmed the indicator function of LPC-O 22:0, implying its key significance in the degranulation process of RBL-2H3 cells.

## 4. Discussion

Due to their potential benefits for human health, dietary polyphenols, such as curcumin (turmeric) and EGCG (green tea), have been a major topic of interest. An increasing number of trials have shown a correlation between dietary polyphenol consumption and a reduction in risk factors for chronic diseases [2,3]. Two typical dietary polyphenols, curcumin and EGCG, were confirmed to show anti-allergic potential in our study. Both curcumin and EGCG significantly suppressed the release of the indicator of degranulation (β-hexosaminidase) and representative pro-inflammatory cytokines (IL-4 and TNF-α) on IgE/antigen-stimulated RBL-2H3 cells. 

Lipids can act as vital intermediates in various cellular communication processes. In this study, the key lipidome remodeling of antigen-stimulated RBL-2H3 cells was further investigated to understand the curcumin/EGCG intervention that underlies the progression of degranulation. Global disturbances in the cellular lipidome were discovered upon IgE/allergen stimulation. Enrichment of 454 significant differential lipids (*p* < 0.05, FDR < 0.05, AG vs. Veh) pinpointed the top lipid categories associated with significant upregulation [i.e., DG(-O)] and downregulation [i.e., (H)BMP, FA, and CAR] after stimulation. Although the progression of IgE-mediated degranulation was revealed by the more evident metabolic changes at 3 h than at 1 h, similar lipidome change patterns were shared during the progression of degranulation. 

Notably, 78% of those significant differential lipids could be regulated upon either curcumin or EGCG intervention. Aside from common features in the improvement of the cellular lipidome by these two typical dietary polyphenols, special lipidome patterns were also found for different intervention groups. Both multivariable and univariate statistical results implied that lipidome regulation in the Cur group (i.e., 10 µM curcumin) was more prominent than that in the EGCG group (i.e., 200 µM EGCG). These lipidomics changes were consistent with the suppression of β-hexosaminidase release by curcumin, which was enhanced compared with EGCG despite their similar performance in decreasing proinflammatory cytokine production. Furthermore, when compared with EGCG, the active intervention by curcumin was highlighted in the immediate phase of the allergic reaction.

### 4.1. DG Metabolism

As the key secondary lipid messengers, DGs were the top lipid categories that underwent significant changes upon degranulation (Figure 3D,E). DGs were significantly increased at the immediate phase of IgE/allergen stimulation, followed by callback at the later phase (Figure 3F). 

The interaction of allergens with IgE–FcεRI complexes results in the formation of signaling complexes that converge on the activation of phospholipase C (PLC) [23]. PLC activation leads to the enzymatic cleavage of phosphoinositol 4,5-bisphosphate (PIP2) into DG and inositol 1,4,5-triphosphate (IP3) [23,24]. IP3 mediates the release of intracellular Ca^2+^ [10,23,24]. DG targets, such as protein kinase C (PKC), Ras guanyl nucleotide-releasing proteins (RasGRP), and the canonical transient receptor potential (TRPC) channel protein, have been shown to be critical in controlling mast cell degranulation [10,23,24]. Consequently, DGs act as the key secondary lipid messengers for transducing signals downstream of receptors. The levels of DGs are tightly associated with the magnitude and duration of the degranulation responses generated. Then, in the AG group, the upregulation of DGs might indicate that antigen–IgE–FcεRI complexes successfully initiated the signaling cascade to activate the process of mast cell degranulation.

The abundance of DGs was regulated by curcumin with an immediate decrease at the early phase of allergen stimulation, indicating that curcumin could inhibit DG-related signal transduction to partially block the process of degranulation. In contrast, DGs could not be recovered by EGCG, suggesting that EGCG was absent from the inhibition of DG-related signal transduction, i.e., antigen stimulation. This was in accordance with the better suppression of mast cell degranulation obtained by curcumin than by EGCG. 

### 4.2. FA Metabolism

With the progression of degranulation, FAs were downregulated from the immediate phase to the later phase and exhibited a prominent decrease upon 3 h of antigen stimulation (Figure 3G). When compared with controls, no significant alterations in FA acyl chain composition in AG were observed at 1 h (Figure 5A), while the abundance of unsaturated fatty acids (incl. MUFA and PUFA) was significantly lower than that of SFA at 3 h (Figure 5B). PUFA metabolism is recognized as an important factor in immune regulation and disease control. The depletion of n-6 PUFAs leads to the production of highly proinflammatory mediators, such as prostaglandins (PGs), thromboxanes (TXs), leukotrienes (LTs), and lipoxins (LXs) [12,25]. As one of the most significant differential lipid classes upon allergen stimulation, the comprehensive regulation of FA abundance and composition may involve degranulation dynamics.

We found that compared to EGCG, curcumin is more potent in upregulating FA metabolism (Figure 4). Three hours of curcumin intervention significantly improved the depletion of MUFA/PUFA (*p* < 0.05, Figure 5C,D). Likewise, the MUFA/PUFA content was replenished by 3 h of EGCG treatment (Figure 5E,F). These changes in the FA profile caused by curcumin/EGCG might contribute to the modification of mast cell gene expression [26]. PPAR-β and -γ have been reported to be expressed in human and murine mast cells and involved in the suppression of mast cell maturation and IgE/antigen-induced production of proinflammatory cytokines [27,28]. At the later phase, the replenishment of PUFAs has been suggested to activate PPAR-γ and change the expression of the antigen response machinery (e.g., FcεRI, Fyn, Lyn, Syk) and degranulation machinery (e.g., calcium channels, vesicle docking molecules) [26]. As a result, mast cells become less susceptible to antigen activation. On the other hand, as the building blocks for lipids, FAs participate in forming membrane phospholipid bilayers and facilitating protein acylation, which is important for the structure and function of membranes/lipid rafts. It has been reported that lipid rafts are especially vital for FcεRI-mediated signal transduction [26]. In IgE/antigen-stimulated cells, FcεRI is quickly recruited to lipid rafts to initiate signaling [26,29]. FcεRI signaling could also be regulated by many lipid raft components [26]. When compared to nonraft regions, the phospholipids in lipid rafts prefer higher levels of saturated fatty acids [30]. Thus, the modification of the FA profile by curcumin/EGCG in the improvement of MUFA/PUFA levels might influence lipid raft function and then reduce FcεRI signaling induced by IgE/antigen, followed by suppression of mediator release from mast cells. 

**Figure 6:**
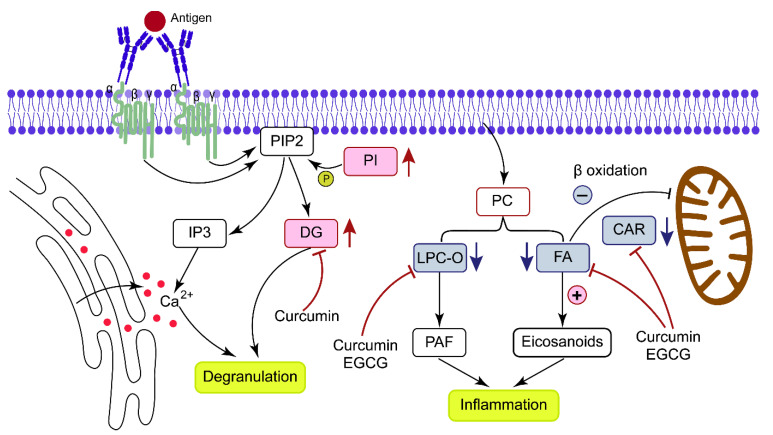
Schematic diagram showing the major rewiring of lipid metabolism.

In addition, CARs, which play a critical role in transporting FAs into mitochondria so they can be oxidized to produce energy, were also defined as one of the top lipid classes with significant changes (Figure 3E). In this study, CARs were found to be decreased in response to IgE/antigen stimulation. The downregulation of CARs may lead to the inhibition of FA β-oxidative, and then promote more FA flow to the pathway of eicosanoic acid synthesis to produce proinflammatory mediators (Figure 6). Decreased FA oxidation and mitochondrial dysfunction have been reported in sensitized mice to support our discoveries [31]. As expected, CARs could be greatly improved by curcumin/EGCG intervention.

### 4.3. BMP Metabolism

In this study, (H)BMPs were found to be decreased upon IgE/antigen stimulation. BMPs are markedly enriched in the inner membranes of late endosomes, particularly lysosomes, and play a key role in lysosomal integrity and function [32,33]. 

BMP-enriched vesicles serve in endosomal-lysosomal tracking and function as docking structures for the activation of lysosomal hydrolytic enzymes [32]. The unique *sn-1:sn-1′* stereoconfiguration of BMP confers its higher resistance to the hydrolytic lysosomal environment [32]. Then, BMP’s negative charge could be retained, facilitating its role as the docking site and essential cofactor for some lysosomal proteins that contain positively charged domains [32,34]. Indeed, the modulation of ABHD6 (i.e., BMP hydrolase) activity has been found to alter the immune response in a murine model of lung inflammation [35]. 

In addition, BMP was reported to be a relatively abundant phospholipid in mast cell-derived extracellular vesicles (EVs), especially degranulated mast cells [36]. Thus, such EVs are derived not only from the plasma membrane or multivesicular bodies but probably also from secretory lysosomes. Mast cells can release EVs constitutively and after IgE-mediated degranulation [36]. In addition to transferring RNA species to other mast cells and containing lipid mediators [37], mast cell-derived EVs exert immune-stimulatory effects on dendritic cells and T/B cells [38,39]. Then, the decrease in (H)BMPs upon IgE/antigen stimulation might also be involved in the release of EVs after IgE-mediated degranulation. Curcumin/EGCG intervention produced favorable effects on the improvement of (H)BMP-related metabolism. 

LPC-O 22:0 was defined as a potential biomarker for its sensitivity to IgE/antigen stimulation and curcumin/EGCG intervention. The remodeling of membrane phospholipids PC by phospholipase A2 (PLA2) generates arachidonic acid (AA) and LPC [12]. Furthermore, LPC is converted to PAF via LPC acetyltransferase (LPCAT) [40]. As one of the key lipid mediators that mast cells abundantly synthesize, PAF signals via the G-protein coupled receptor (GPCR) (PAF receptor, PAFR), which initiates a signaling cascade [12]; then degranulation and an enhancement in inflammation are triggered [12]. The recovery of the potential biomarker LPC by curcumin/EGCG thus indicated that their effective intervention may involve the inhibition of PAF generation. 

Certainly, these lipidome changes might be the result of the development of protein–polyphenol complexes with possibly lower allergenic potential [5,6], as well as more sophisticated and comprehensive anti-allergy mechanisms. Following that, a growing number of studies have demonstrated that mast cell synthesis and metabolism of lipid mediators, in turn, influence cellular processes [9,11]. The role of lipids in the pathogenesis of allergic disease has long been studied. Our study indicated that a potency was observed with curcumin than with EGCG in the disturbance of lipid metabolism, in accordance with the superior effects of curcumin observed when compared with EGCG in the suppression of the degranulation process. A considerable similarity between curcumin intervention and EGCG was discovered in their lipidome remodeling patterns. Our study confirmed the significance of DG, FA, BMP, and LPC metabolism for IgE/antigen stimulation and subsequent curcumin/EGCG intervention. Both changes in lipid response and composition patterns indicated that lipids influence the degranulation process via multiple mechanisms, including (i) producing highly proinflammatory mediators, (ii) mediating intracellular signaling cascades by acting as second messengers, (iii) activating a diverse family of receptors, and (iv) forming structural support platforms (lipid rafts) and extracellular vesicles. 

The goal of this study is to describe curcumin/EGCG-induced lipidome change in order to give a novel perspective on curcumin/EGCG participation in anaphylaxis. It should be noted, however, that research into the process is currently restricted. The underlying mechanisms that influence lipid alterations by putative protein–polyphenol complexes with possibly decreased allergenic potential, as well as the subsequent effects of lipid changes on allergy reactions, require additional exploration. Further research will be needed, including expanding the sampling distribution throughout the degranulation course, tracking the effects of curcumin and EGCG in vivo, and a combined analysis including transcriptomic, biochemical, and immunological results.

## 5. Conclusions

Two typical dietary polyphenols, curcumin and EGCG, were confirmed to show anti-allergic potential in the present study. Both curcumin and EGCG significantly suppressed the release of β-hexosaminidase, IL-4, and TNF-α from IgE/antigen-stimulated RBL-2H3 cells. As compared to the alteration pattern associated with IgE/antigen-stimulated degranulation (Veh vs. AG), the lipidome modulation by curcumin/EGCG could be identified (Cur vs. AG and EGCG vs. AG). Comprehensive lipidomics analysis revealed that the ability to disturb lipid metabolism was stronger with curcumin than EGCG, in accordance with the superior ability of curcumin to suppress the degranulation process. These key lipidome disturbances provide novel insights into the effects of curcumin/EGCG intervention underlying the progression of degranulation. Our findings open the possibility of preventing immediate allergic reactions via antigen-stimulated mast cells in vitro and will help guide future attempts to use dietary polyphenols.

## Figures and Tables

**Table 1:** Statistical information of biomarker candidates.

Compound	Fatty Acids	Veh vs. AG				Curcumin vs. AG				EGCG vs. AG			
		*p*	FDR	AG/Veh		*p*	FDR	Cur/AG		*p*	FDR	EGCG/AG	
				Ratio-1 h	Ratio-3 h			Ratio-1 h	Ratio-3 h			Ratio-1 h	Ratio-3 h
CAR 24:0	24:0	4.51 × 10^−10^	1.27 × 10^−8^	0.611	0.522	9.38 × 10^−4^	2.64 × 10^−3^	1.159	1.240	3.52 × 10^−1^	4.63 × 10^−1^	0.849	1.241
CAR 24:1	24:1	1.77 × 10^−10^	6.10 × 10^−9^	0.580	0.582	1.48 × 10^−4^	5.12 × 10^−4^	1.103	1.350	6.81 × 10^−2^	1.27 × 10^−1^	0.861	1.220
DG 38:4	18:0_20:4	3.90 × 10^−14^	1.82 × 10^−11^	11.091	2.447	5.23 × 10^−1^	6.16 × 10^−1^	0.913	1.229	1.44 × 10^−5^	1.11 × 10^−4^	1.495	1.743
DG 40:5	18:0_22:5	1.64 × 10^−13^	4.88 × 10^−11^	7.501	1.702	1.56 × 10^−1^	2.37 × 10^−1^	0.969	1.320	1.16 × 10^−4^	6.10 × 10^−4^	1.372	1.397
DG 40:6	18:0_22:6	2.62 × 10^−13^	4.88 × 10^−11^	7.822	1.649	1.54 × 10^−2^	3.22 × 10^−2^	1.025	1.351	1.08 × 10^−4^	5.81 × 10^−4^	1.399	1.324
HBMP 58:8	18:1_18:1_22:6	2.76 × 10^−9^	5.47 × 10^−8^	0.648	0.643	3.19 × 10^−4^	1.00 × 10^−3^	1.081	1.278	2.01 × 10^−1^	3.00 × 10^−1^	1.080	0.992
HBMP 62:13	18:1_22:6_22:6	1.72 × 10^−11^	1.11 × 10^−9^	0.582	0.529	7.29 × 10^−1^	7.93 × 10^−1^	0.855	1.158	6.06 × 10^−2^	1.14 × 10^−1^	1.018	1.111
HBMP 64:16	20:4_22:6_22:6	3.66 × 10^−12^	3.41 × 10^−10^	0.569	0.478	2.99 × 10^−3^	7.68 × 10^−3^	0.725	1.041	5.32 × 10^−2^	1.02 × 10^−1^	0.999	1.140
LPC-O 22:0	O-22:0	2.52 × 10^−11^	1.30 × 10^−9^	0.658	0.581	1.35 × 10^−7^	2.42 × 10^−6^	1.209	1.477	5.04 × 10^−4^	1.90 × 10^−3^	1.045	1.163
LPC-O 24:1	O-24:1	7.71 × 10^−10^	1.99 × 10^−8^	0.584	0.524	8.44 × 10^−9^	7.14 × 10^−7^	1.287	1.517	2.89 × 10^−2^	6.04 × 10^−2^	1.060	1.052
PEtOH 34:1	16:0_18:1	2.64 × 10^−9^	5.34 × 10^−8^	2.239	4.310	1.86 × 10^−1^	2.71 × 10^−1^	0.670	1.121	1.98 × 10^−6^	2.49 × 10^−5^	1.728	1.197
PEtOH 36:1	18:0_18:1	5.14 × 10^−8^	6.47 × 10^−7^	1.511	2.358	1.09 × 10^−1^	1.77 × 10^−1^	0.696	1.047	8.64 × 10^−5^	4.82 × 10^−4^	1.524	1.156
PG 36:4	16:0_20:4	1.79 × 10^−11^	1.11 × 10^−9^	1.505	1.650	3.32 × 10^−4^	1.03 × 10^−3^	0.795	1.007	3.62 × 10^−7^	6.02 × 10^−6^	1.088	1.328
PG 38:4	18:0_20:4	5.71 × 10^−12^	4.83 × 10^−10^	3.010	3.106	9.49 × 10^−4^	2.66 × 10^−3^	0.723	0.993	3.01 × 10^−7^	5.22 × 10^−6^	1.341	1.634
PI 36:5	16:0_20:5	1.42 × 10^−12^	1.47 × 10^−10^	2.175	2.374	2.15 × 10^−5^	1.02 × 10^−4^	1.043	1.197	9.42 × 10^−1^	9.68 × 10^−1^	0.864	1.135
PI 42:9	20:4_22:5	7.28 × 10^−13^	9.68 × 10^−11^	3.257	2.087	9.68 × 10^−5^	3.62 × 10^−4^	0.759	1.061	1.86 × 10^−2^	4.14 × 10^−2^	0.939	1.321
PI-O 36:4	O-16:0_20:4	1.72 × 10^−10^	6.10 × 10^−9^	1.691	1.759	9.41 × 10^−2^	1.57 × 10^−1^	0.905	1.012	3.72 × 10^−1^	4.83 × 10^−1^	0.859	1.225
PI-O 38:6	O-16:0_22:6	5.88 × 10^−10^	1.61 × 10^−8^	1.556	1.658	2.83 × 10^−3^	7.32 × 10^−3^	1.035	1.127	9.71 × 10^−2^	1.69 × 10^−1^	0.868	0.999
PI-O 40:7	O-18:1_22:6	1.26 × 10^−12^	1.47 × 10^−10^	1.546	1.894	4.01 × 10^−1^	5.09 × 10^−1^	1.013	1.010	3.18 × 10^−2^	6.56 × 10^−2^	0.945	0.924

## Data Availability

The datasets generated and/or analysed during the current study are available in the Metabolomics Workbench (https://www.metabolomicsworkbench.org/, accessed on 1 January 2023) repository (Study ID ST002384, DatatrackID: 3356 and Project DOI: http://dx.doi.org/10.21228/M8DH62, accessed on 1 January 2023).

## Supplementary Materials

The following supporting information can be downloaded at: https://www.mdpi.com/article/10.3390/metabo13050628/s1, Supplementary data associated with this article can be found in the online version. Materials and Methods of Chemicals and Reagents, Lipidome Extraction, and Lipidomics Analysis by UPLC-Q-Exactive MS. Results of Analytical Performance of Lipid Profiling [19,20,21]; Figure S1: Characteristics of the experimental model; Figure S2: Cell morphology; Figure S3: Lipidome profiling; Figure S4: The distributions of %number for identified lipid species; Figure S5: Analytical Performance of Lipid Profiling; Figure S6: The comparison between AG and Veh groups; Figure S7: The comparison between AG and curcumin groups; Figure S8: The comparison between AG and EGCG groups; Figure S9: LPC-O changes associated with acyl chain composition; Figure S10: Identification of the potential biomarker LPC-O 22:0; Table S1: Statistical results of identification; Table S2: Information of identified lipids; Table S3: Statistical information of differential lipids changed by degranulation; Table S4: Changes in the content and composition of important lipids.
