# Supplementary material for: Anti-Allergic Effect of Dietary Polyphenols Curcumin and Epigallocatechin Gallate via Anti-Degranulation in IgE/Antigen-Stimulated Mast Cell Model: A Lipidomics Perspective
Source: Metabolites. 2023 May 5;13(5):628. doi: 10.3390/metabo13050628 (PMC10223132; doi:10.3390/metabo13050628)
Supplement: Supplementary file 1 [file metabolites-13-00628-s001.zip › Supporting Information.pdf]

Supporting Information

Anti-allergic Effect of Dietary Polyphenols Curcumin and Epigallocatechin Gallate via Anti-degranulation in IgE/Antigen-stimulated Mast Cell Model: A Lipidomics Perspective

Jun Zeng[1,3,*,†], Jingwen Hao[1,†], Zhiqiang Yang[1,†], Chunyu Ma[1], Longhua Gao[1], Yue Chen[4], Guiling Li[1,3], Jia Li[2,*]

[1] College of Ocean Food and Biological Engineering, Jimei University, Xiamen 361021, China

[2] Key Laboratory of Tea Biology and Resources Utilization, Ministry of Agriculture, Tea Research Institute, Chinese Academy of Agricultural Sciences, Hangzhou 310008, China

[3] Xiamen Key Laboratory of Marine Functional Food, Xiamen 361021, China

[4] The Affiliated Stomatology Hospital, School of Medicine, Zhejiang University, Hangzhou 310000, China

[†]: Equal contribution

*: Corresponding authors

Dr. Jun Zeng, Tel.: 86-592-6181487. E-mail: junzeng@jmu.edu.cn.

Dr. Jia Li, Tel.: 86-571-86650637. E-mail: jiali1986@tricaas.com.

Content:

1. MATERIALS AND METHODS of Chemicals and Reagents

2. MATERIALS AND METHODS of Lipidome Extraction

3. MATERIALS AND METHODS of Lipidomics Analysis by UPLC-Q-Exactive MS

4. RESULTS of Analytical Performance of Lipid Profiling

5. Figure S1. Characteristics of the experimental model.

6. Figure S2. Cell morphology.

7. Figure S3. Lipidome profiling.

8. Figure S4. The distributions of %number for identified lipid species.

9. Figure S5. Analytical Performance of Lipid Profiling.

10. Figure S6. The comparison between AG and Veh groups.

11. Figure S7. The comparison between AG and curcumin groups.

12. Figure S8. The comparison between AG and EGCG groups.

13. Figure S9. LPC-O changes associated with acyl chain composition.

14. Figure S10. Identification of the potential biomarker LPC-O 22:0.

15. Table S1. Statistical results of identification.

16. Table S2. Information of identified lipids.

17. Table S3. Statistical information of differential lipids changed by degranulation.

18. Table S4. Changes in the content and composition of important lipids.

MATERIALS AND METHODS

Chemicals and Reagents

Hyclone Essential Medium with Earle's Balanced Salts (MEM/EBSS), fetal bovine serum (FBS) and Gibco penicillin-streptomycin solution were respectively purchased from Cytiva (USA), Gemini (USA) and ThermoFisher Scientific (USA). Tyrode's solution was purchased from Solarbio (China). Monoclonal anti-dinitrophenyl antibody produced in mouse (Anti-DNP-IgE), 4-methylumbelliferyl-N-acetyl-β-D-glucosaminide, 3-(4,5-dimethylthiazol-2-yl)-2,5-diphenyl-tetrazolium bromide (MTT), curcumin and epigallocatechin gallate (EGCG) were purchased from Sigma-Aldrich (USA). Dinitrophenyl-bovine serum albumin (DNP-BSA) was purchased from LGC, Biosearch Technologies (UK). Prior to the experiment, the stock solutions of Anti-DNP-IgE and DNP-BSA were prepared in Dulbecco's phosphate-buffered saline (DPBS), and the stock solutions of curcumin and EGCG were prepared in dimethyl sulfoxide (DMSO).

Methanol, acetonitrile and isopropanol of liquid chromatography grade were purchased from Merck (Germany). Tert-butyl methyl ether (MTBE), ammonium acetate (> = 98%) were purchased from Sigma-Aldrich (USA). Ultrapure water was prepared by a Millipore Milli-Q system (USA). Lipid standards, namely, phosphatidylcholine (PC) (19:0/19:0), phosphatidylethanolamine (PE) (15:0/15:0), lysophospatidylcholine (LPC) (19:0), sphingomyelin (SM)(d18:1/12:0), triacylglycerol (TG) (15:0/15:0/15:0), ceramide (Cer) (d18:1/17:0), palmitic acid (fatty acid (FA) (16:0))-d3 and stearic acid (FA(18:0))-d3, were purchased from Avanti Polar Lipids (USA), Cambridge Isotope Laboratories (USA) and Sigma-Aldrich.

Lipidome Extraction

Four independent biological replicates of RBL-2H3 cells were prepared for each group at each time point in this lipidomics study. The cell medium was completely aspirated after sample collection, followed by washing with Dulbecco's phosphate-buffered saline (DPBS) solution and inactivation with liquid nitrogen immediately.

Each cell sample was then spiked with 1 mL of methanol containing internal standards (1.12 μg/mL of phosphatidylcholine (PC) (19:0/19:0), 0.92 μg/mL of phosphatidylethanolamine (PE) (15:0/15:0), 0.87 μg/mL of lysophospatidylcholine (LPC) (19:0), 0.87 μg/mL of sphingomyelin (SM) (d18:1/12:0), 0.67 μg/mL of triacylglycerol (TG) (15:0/15:0/15:0), 0.47 μg/mL of ceramide (Cer) (d18:1/17:0), 1 μg/mL of palmitic acid (fatty acid (FA) (16:0))-d3, and 1 μg/mL of stearic acid (FA (18:0))-d3). Cells were scraped from the culture dish and transferred into a 5 mL Eppendorf tube. Subsequently, 2.5 mL of tert-butyl methyl ether (MTBE) was added to each tube. After 30 min vortex, 750 μL of Milli-Q water was added to produce two phases. The upper layer, that is hydrophobic phase, was collected after centrifugation (10,000 rpm for 15 min at 6 °C) and freeze-dried in a refrigerated CentriVap concentrator (Labconco, USA), followed by storage at -80 °C until analysis.

Lipidomics Analysis by UPLC-Q-Exactive MS

LC-MS based lipidomics profiling was conduct using an UltiMate 3000 UPLC system (Thermo, USA) coupled with a quadrupole Orbitrap mass spectrometer (Q-Exactive, Thermo, USA), adopted from our previously published method[1-3].

Lipid extracts were dissolved and separated using a BEH C8 column (2.1 × 100 mm, 1.7

μm) (Waters, USA). Gradient elution was started with 32% solvent B (isopropanol/acetonitrile (9:1, v/v) with 10 mM ammonium acetate) and 68% solvent A (acetonitrile/water (6:4, v/v) with 10 mM ammonium acetate), which was maintained for 1.5 min, followed by linear increase to 85% B during next 14 min. Solvent B was further linearly increased to 97% in 0.1 min and kept for 2.4 min. Then, the gradient was quickly changed back to 32% B in 0.1 min and equilibrated for another 1.9 min until the next injection. The elution was maintained for 20 min for each injection with a flow rate of 0.26 mL/min. The sample tray was set at 10 °C, and the injection volume was 5 μL.

Full-scan MS for lipid profiling and data-dependent MS/MS (ddMS2) for lipid identification was acquired in both positive and negative electrospray (ESI) ion modes. When heated electrospray (HESI) was applied in the positive ion mode, parameters were set as follows: spray voltage, 3.5 kV; capillary temperature, 325 °C; sheath gas flow rate, 50; aux gas flow rate, 15; S-lens RF level, 50; AGC (automatic gain control) target, 3 × 106 ions capacity for full scan MS and 1 × 105 ions capacity for ddMS2; maximum IT (injection time), 100 ms for full scan MS and 50 ms for ddMS2; normalized collision energy (NCE), 25 and 35; TopN (N, the number of top most abundant ions for fragmentation), 10; resolution, 140, 000 for full scan MS and 35, 000 for ddMS2; scan range, 200-1800 *m/z*. Negative ESI setting of spray voltage was 3.0 kV. Other parameters were identical to those used in the positive mode.

Quality control (QC) samples, generated by pooling equal aliquots of lipid extracts from each sample, were prepared as real samples and regularly inserted into the analysis sequence to monitor the robustness of lipidomic analysis.

RESULTS

Analytical Performance of Lipid Profiling

The reliability and robustness of acquired lipidomics data was investigated by evaluating QC samples. Unsupervised principal component analysis (PCA) shown that QC samples from positive and negative analysis modes cluster closely in each score plots (Figure S5A and B). The deviation of QCs was within 2 times of standard deviation (SD) (Figure S5C and D). The distributions of %RSD for QC samples indicated that lipids with %RSD lower than 30% accounted for 97.16% (positive mode, Figure S5E) and 98.54% (negative mode, Figure S5F) of sum of lipid number. These results were confirmed to be satisfactory for complex biological samples.

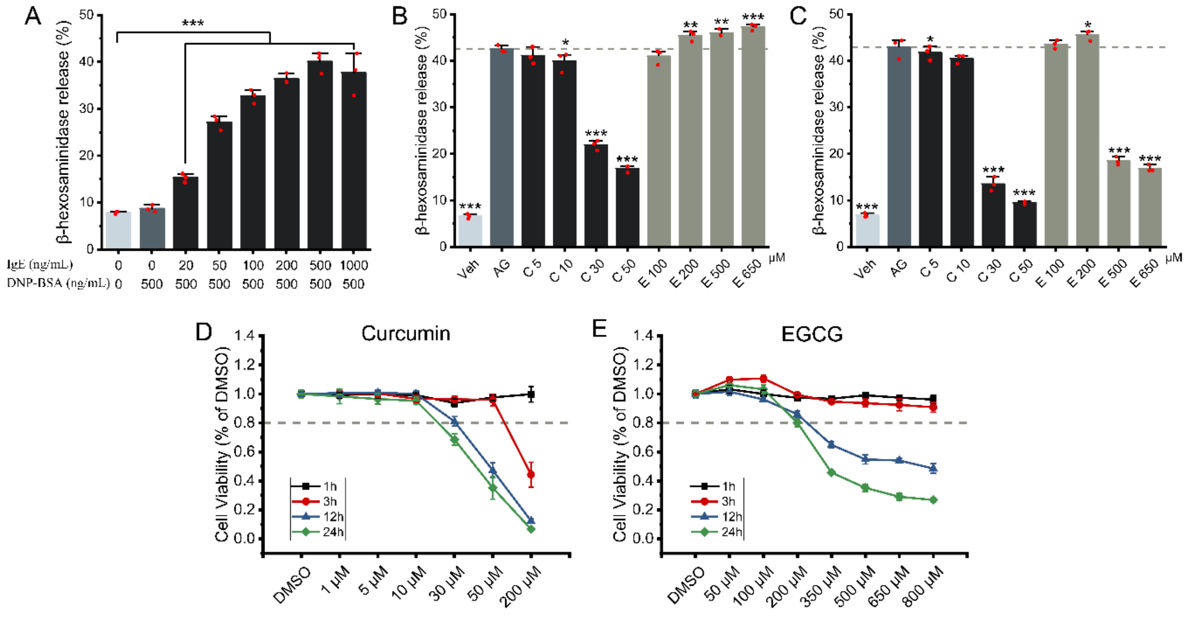

Figure S1. Characteristics of the experimental model. (A) Effects of IgE concentration on degranulation of RBL-2H3 cells. To compare with the modeling way in which DNP-BSA and curcumin/EGCG were co-incubated (Figure 2), an alternative operation was performed in our previous modeling. IgE-sensitized cells were first treated with curcumin/EGCG for 1h and 3h, respectively. The treated cells were subsequently stimulated with DNP-BSA for 1h to perform the β-hexosaminidase release assay. For this modeling operation, (B) and (C) present β-hexosaminidase release for 1h and 3h of curcumin/EGCG treatment, respectively. To achieve better inhibition of β-hexosaminidase release, 200 ng/mL of anti-DNP-IgE for sensitization and the co-incubation of DNP-BSA and curcumin/EGCG for stimulation (Figure 1D and E) were defined in our present modeling. (D) and (E) Cell viability for curcumin and EGCG, respectively. In order to evaluate the cytotoxicity of curcumin/EGCG on RBL-2H3 cells, the cells were treated with different concentrations of curcumin/EGCG for different duration of time. All data are presented as the mean ± SD. *: $0.01 < p < 0.05$, **: $0.001 < p < 0.01$, ***: $p < 0.001$.

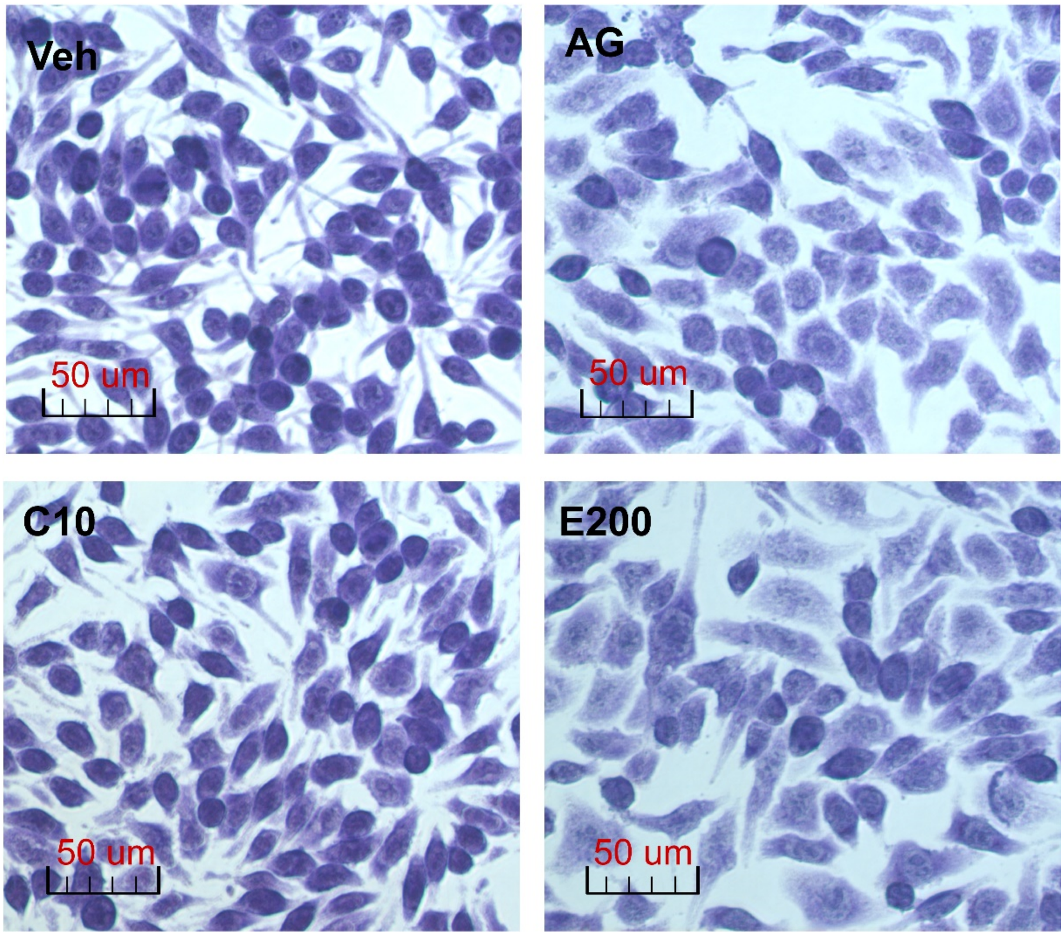

Figure S2. Cell morphology. Veh: vehicle control group; AG: IgE/antigen stimulation group; C10: intervention group in which IgE/antigen stimulated cells were treated by 10 μM of curcumin; E200: intervention group in which IgE/antigen stimulated cells were treated by 200 μM of EGCG.

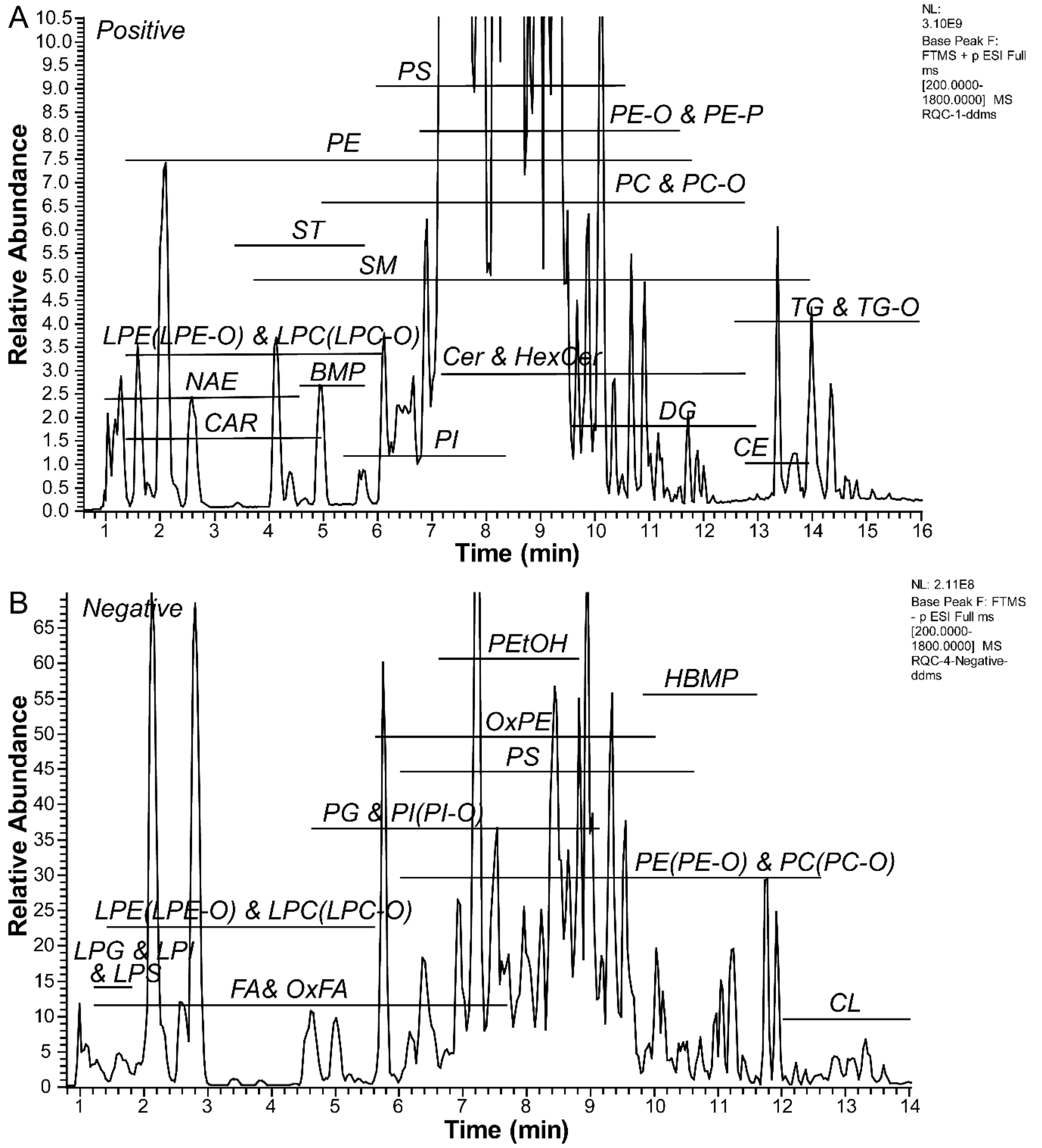

Figure S3. Lipidome profiling. Base peak chromatography (BPC) of pooled cell extract acquired using LC-MS-based lipidomics approach in ESI positive (A) and negative mode (B), respectively.

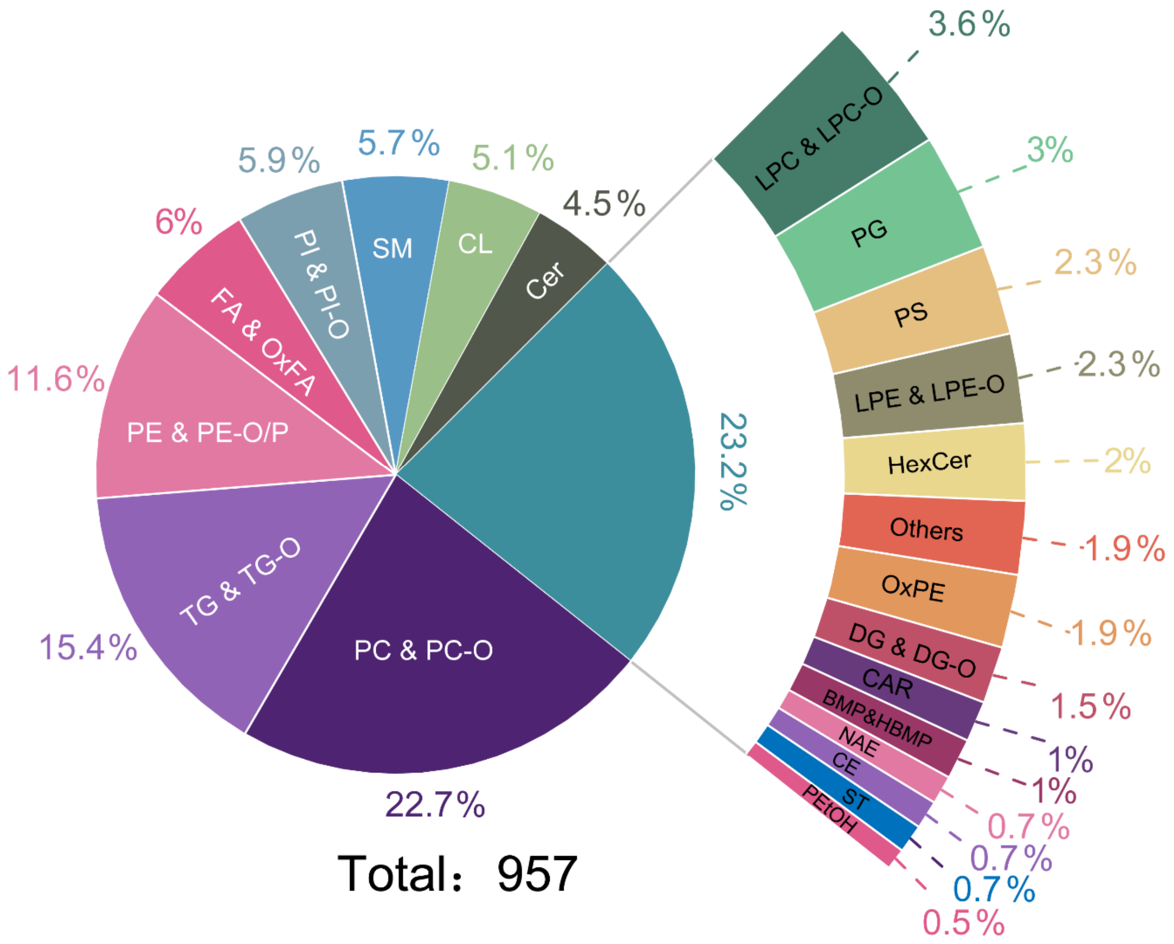

Figure S4. The distributions of %number for identified lipid species.

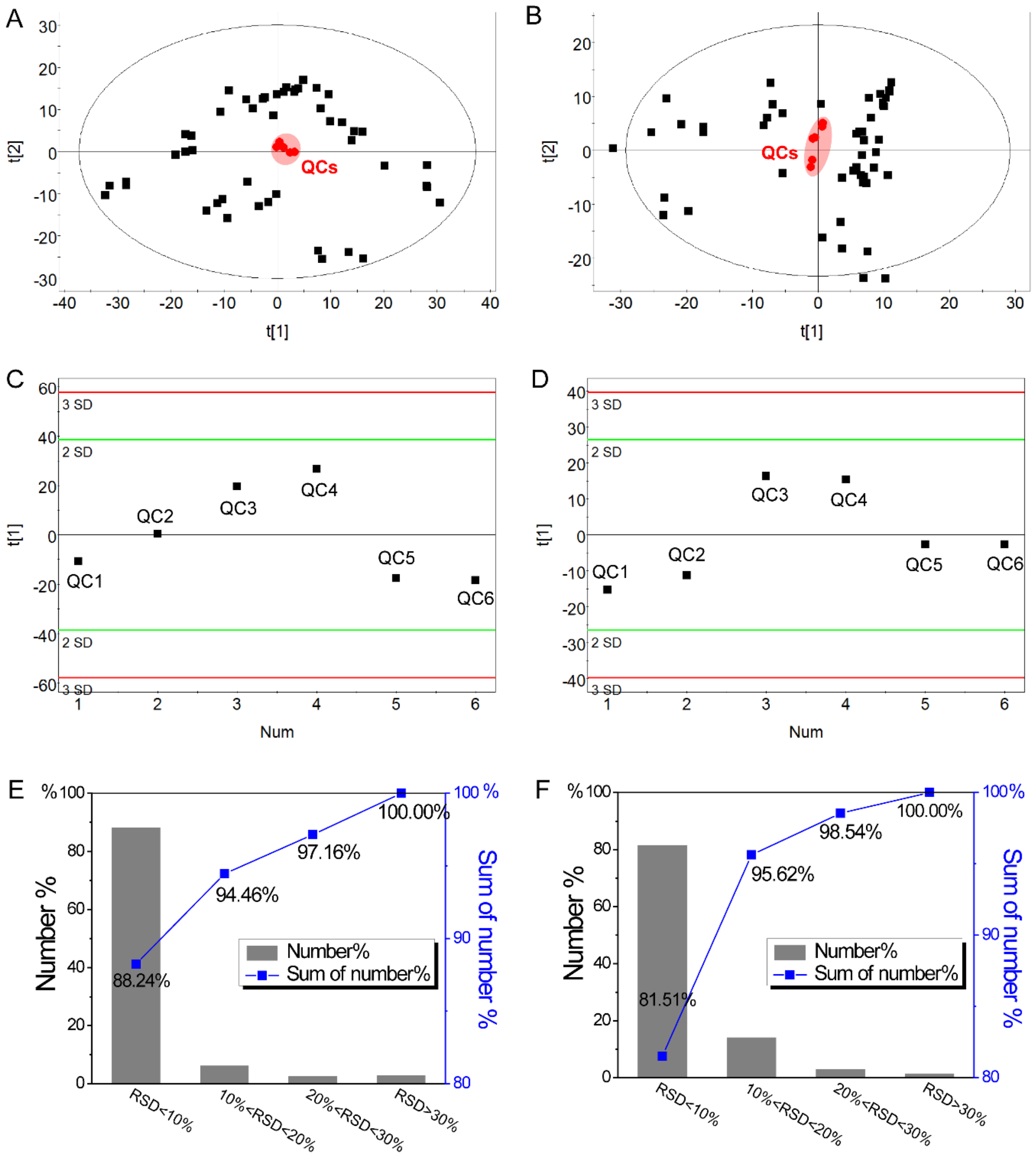

Figure S5. Analytical Performance of Lipid Profiling. (A) and (B) are PCA score plots for all samples from positive and negative analysis mode, respectively. (C) and (D) are PCA score plots for QC samples from positive and negative analysis mode, respectively. (E) and (F) indicate the distributions of %RSD for lipid species among all QC samples from positive and negative mode, respectively.

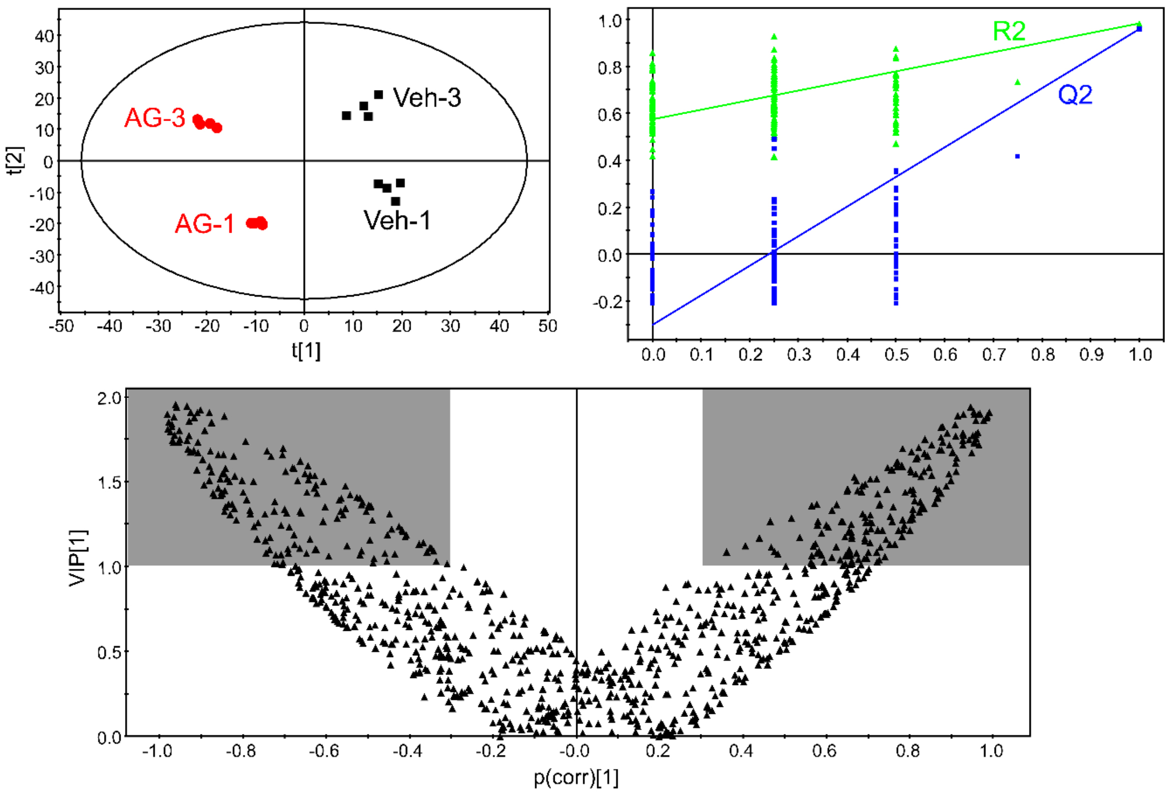

Figure S6. Comparison between AG and Veh groups. (A) PLS-DA score plot for the classification of vehicle (Veh-1 and Veh-3) and IgE/antigen stimulation (AG-1 and AG-3) groups at both 1h and 3h. R2X = 0.572; R2Y = 0.983; Q2 = 0.96. (B) Validation of PLS-DA model. This PLS-DA model was cross-validated without overfitting by 200 permutations. R2 = (0, 0.567); Q2 = (0, -0.315). (C) V-plot of the first principal component. Lipids with VIP [1] > 1 and |p(corr)| > 0.3 were spotted on the V-plot.

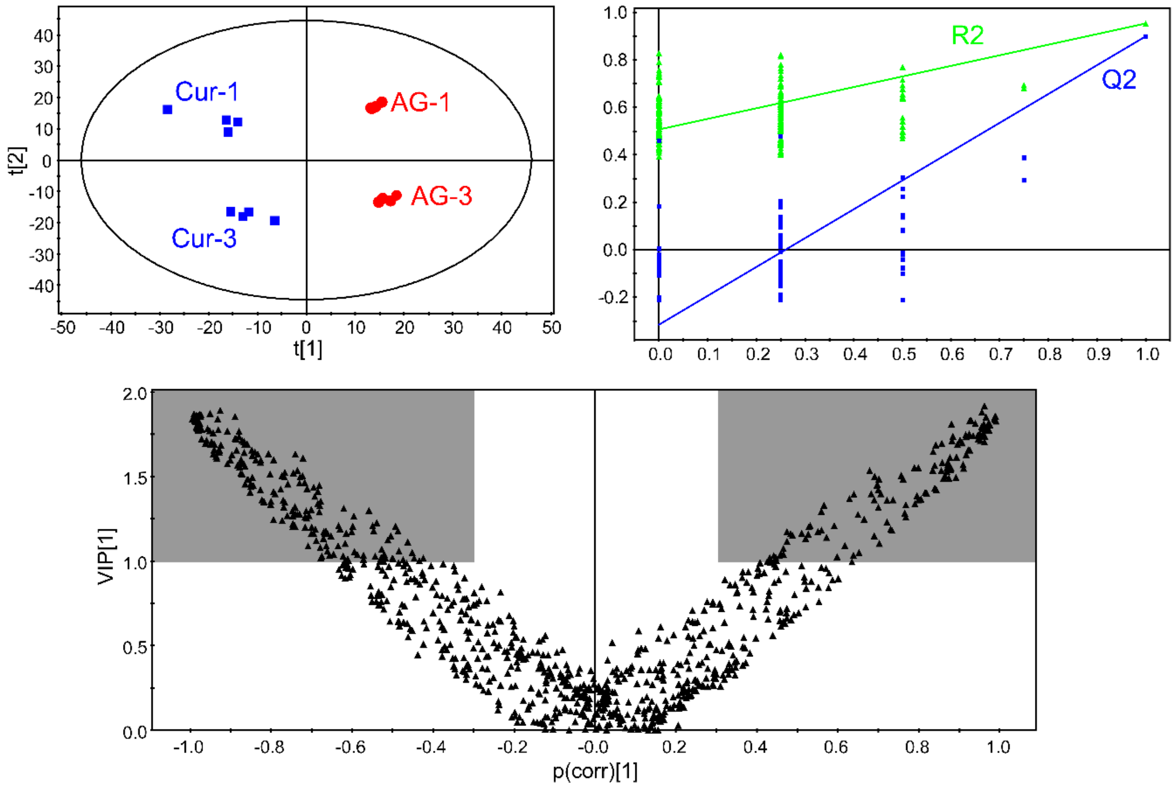

Figure S7. The comparison between AG and curcumin groups. (A) PLS-DA score plot for the classification of IgE/antigen stimulation (AG-1 and AG-3) and curcumin (Cur-1 and Cur-3) groups at 1h and 3h. R2X = 0.607; R2Y = 0.954; Q2 = 0.899. (B) Validation of PLS-DA model. This PLS-DA model was cross-validated without overfitting by 200 permutations. R2 = (0, 0.508); Q2 = (0, -0.315). (C) V-plot of the first principal component. Lipids with VIP [1] > 1 and |p(corr)| > 0.3 were spotted on the V-plot.

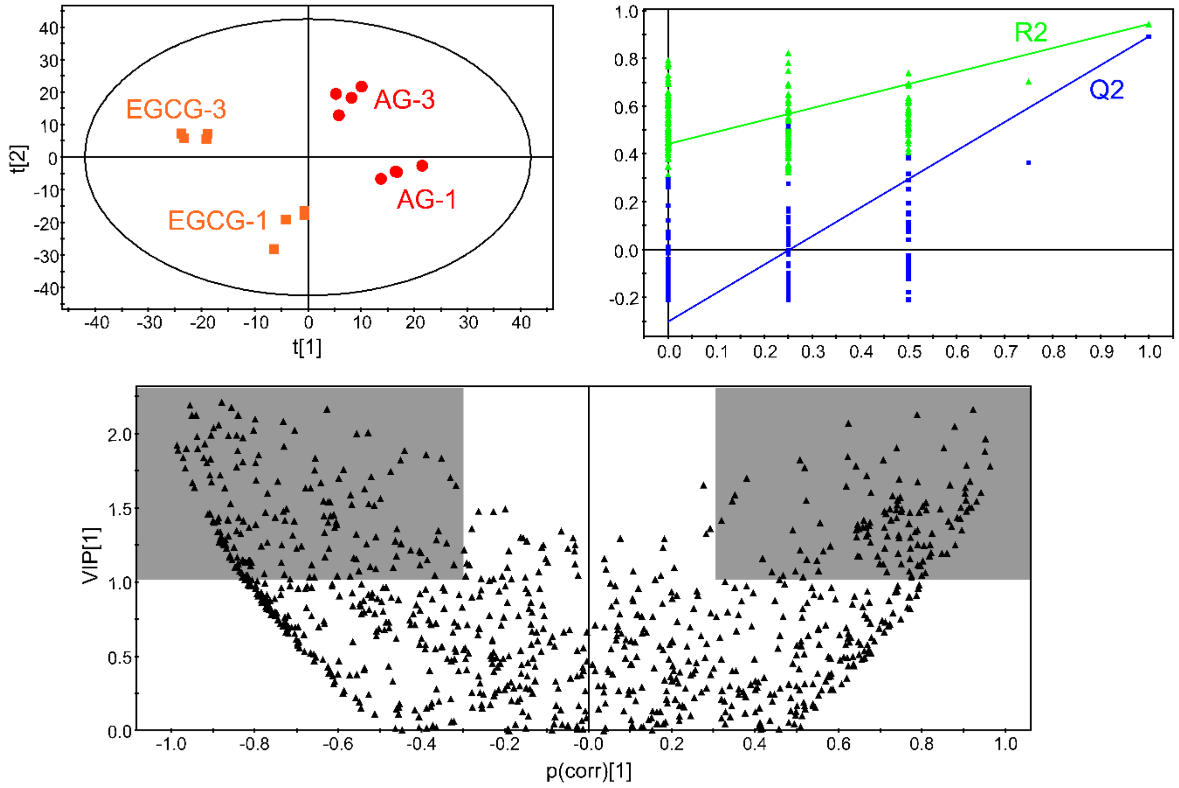

Figure S8. The comparison between AG and EGCG groups. (A) PLS-DA score plot for the classification of IgE/antigen stimulation (AG-1 and AG-3) and EGCG (EGCG-1 and EGCG-3) groups at both 1h and 3h. R2X = 0.567; R2Y = 0.943; Q2 = 0.89. (B) Validation of PLS-DA model. This PLS-DA model was cross-validated without overfitting by 200 permutations. R2 = (0, 0.442); Q2 = (0, -0.301). (C) V-plot of the first principal component. Lipids with VIP [1] > 1 and |p(corr)| > 0.3 were spotted on the V-plot.

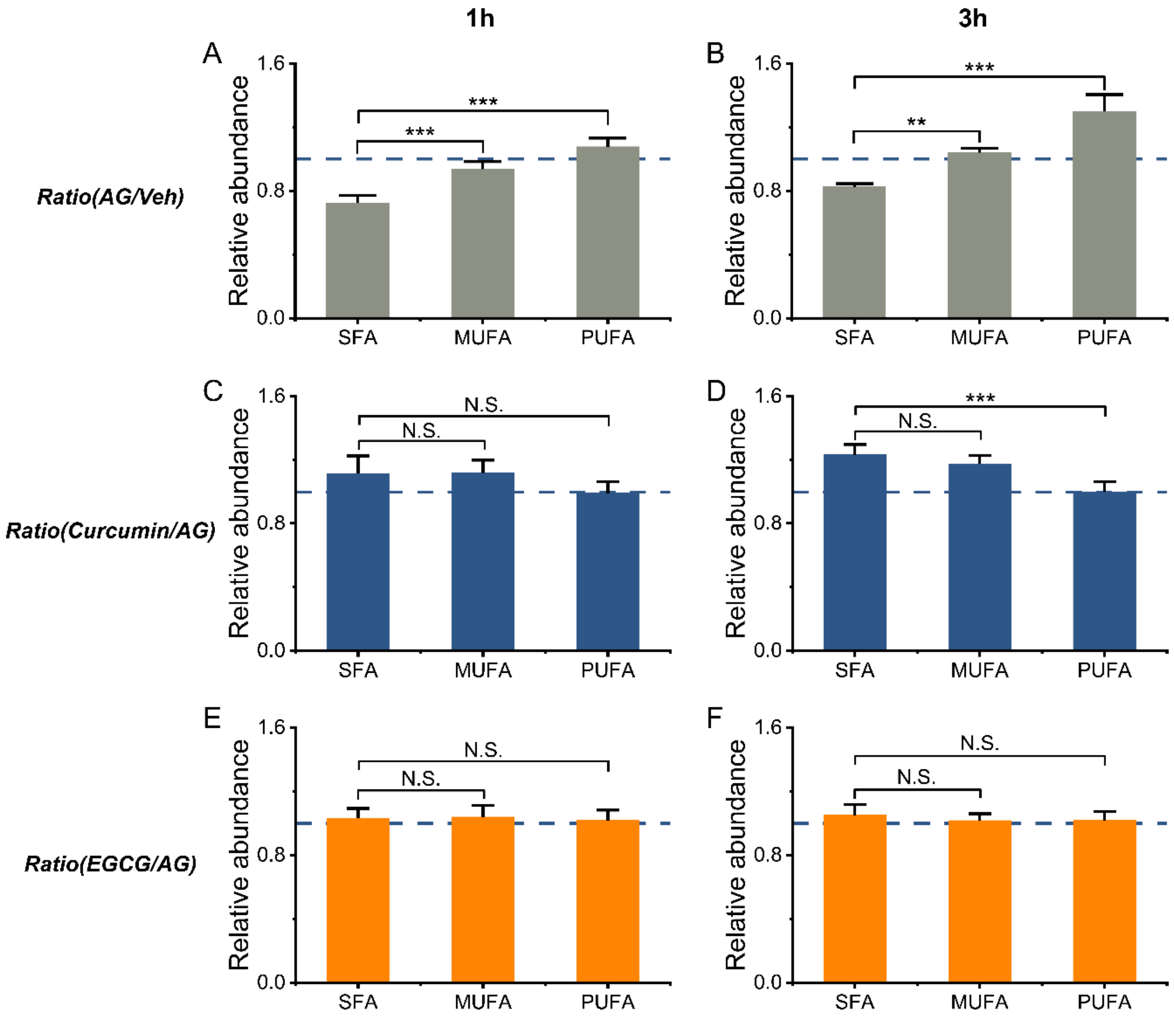

Figure S9. LPC-O changes associated with acyl chain composition. Each column is presented as the mean ± SD. *: $0.01 < p < 0.05$, **: $0.001 < p < 0.01$, ***: $p < 0.001$, N.S.: no significance.

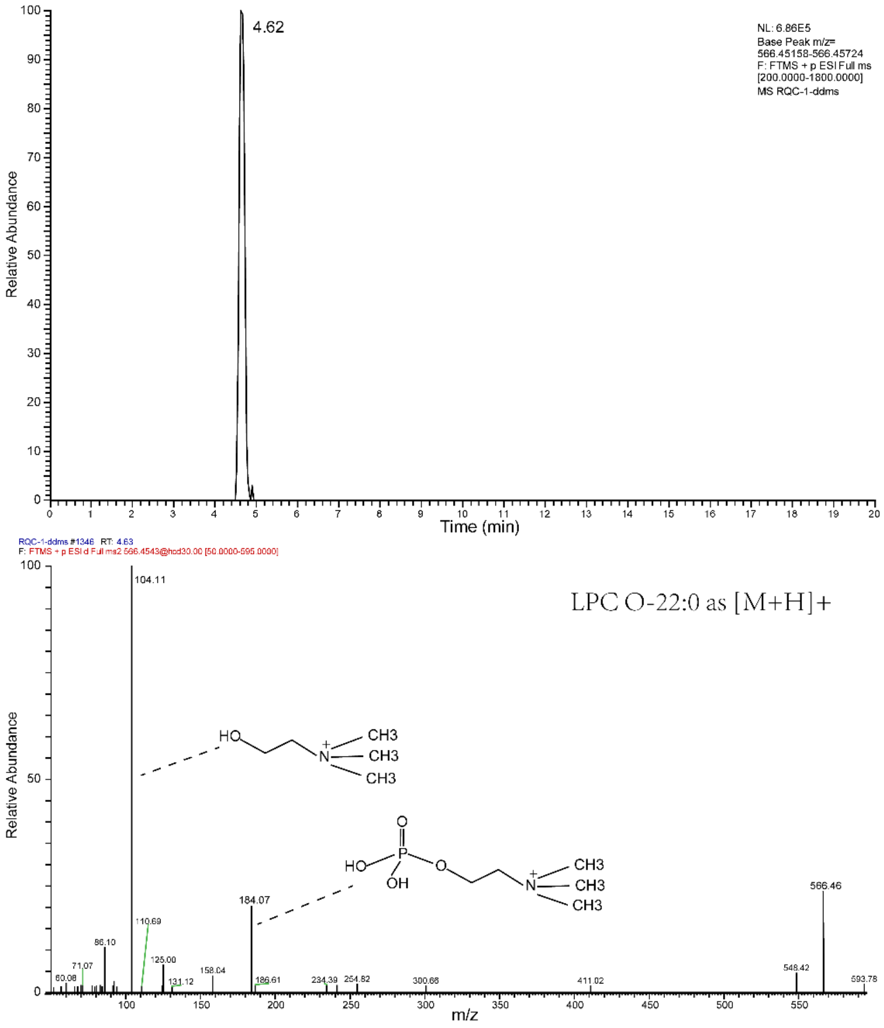

Figure S10. Identification of the potential biomarker LPC-O 22:0.

REFERENCES

1. Zeng, J.; Li, J.L.; Liu, S.S.Y.; Yang, Z.Q.; Zhong, Y.; Chen, X.M.; Li, G.L.; Li, J. Lipidome disturbances in preadipocyte differentiation associated with bisphenol A and replacement bisphenol S exposure. *Sci. Total Environ.* **2021**, *753*, 10, doi:10.1016/j.scitotenv.2020.141949.
2. Zeng, J.; Liu, S.; Cai, W.; Jiang, H.; Lu, X.; Li, G.; Li, J.; Liu, J. Emerging lipidome patterns associated with marine Emiliania huxleyi-virus model system. *Sci. Total Environ.* **2019**, *688*, 521-528, doi:10.1016/j.scitotenv.2019.06.284.
3. Li, J.; Yuan, H.B.; Rong, Y.T.; Qian, M.C.; Liu, F.Q.; Hua, J.J.; Zhou, Q.H.; Deng, Y.L.; Zeng, J.; Jiang, Y.W. Lipid metabolic characteristics and marker compounds of ripened Pu-erh tea during pile fermentation revealed by LC-MS-based lipidomics. *Food Chem.* **2023**, *404*, 12, doi:10.1016/j.foodchem.2022.134665.
